# Frequency shift of the Bragg and Non-Bragg backscattering from periodic water wave

**DOI:** 10.1038/srep31588

**Published:** 2016-08-17

**Authors:** Biyang Wen, Ke Li

**Affiliations:** 1 School of Electronic Information, Wuhan University, Wuhan, 430072, China

## Abstract

Doppler effect is used to measure the relative speed of a moving target with respect to the radar, and is also used to interpret the frequency shift of the backscattering from the ocean wave according to the water-wave phase velocity. The widely known relationship between the Doppler shift and the water-wave phase velocity was deduced from the scattering measurements data collected from actual sea surface, and has not been verified under man-made conditions. Here we show that this ob- served frequency shift of the scattering data from the Bragg and Non-Bragg water wave is not the Doppler shift corresponding to the water-wave phase velocity as commonly believed, but is the water-wave frequency and its integral multiple frequency. The power spectrum of the backscatter from the periodic water wave consists of serials discrete peaks, which is equally spaced by water wave frequency. Only when the water-wave length is the integer multiples of the Bragg wave, and the radar range resolution is infinite, does the frequency shift of the backscattering mathematically equal the Doppler shift according to the water-wave phase velocity.

The Doppler effect is a well-known phenomenon, which states the frequency of a wave changes according to the relative velocity of the source and the observer. The effect is widely accepted, and has been used in radar, sonar and sensors to measure the relative speed of a moving target^1^,^2^,^3^.

Doppler effect is also used to interpret the frequency shift of the HF radio wave’s backscatter from the moving ocean wave. In 1955, Crombie^4^ observed sea echo with a HF radar at 13.5 *MHz*. The radar sea-echo spectra contain two dominant peaks shift from the incident radio frequency. Crombie explained that the two dominant peaks were Bragg scatter, coherently backscatter comes from ocean waves half the radar wavelength *λ*, propagating radially toward, or away from the radar. And the frequency shift 0.38 *Hz*, is the Doppler shift corresponding to the phase velocity of the Bragg wave. The frequency shift of the Bragg scatter is named as Bragg frequency *f*_*B*_ afterwards.

Crombie mentioned there was a subsidiary peak at a frequency of 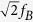 in the sea echo spectrum, and Crombie thought it is due to waves having a length twice the length of the Bragg wave with the phase velocity 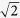 times the Bragg wave. Barrick^5^ attributed the subsidiary peak with the frequency of 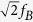 to some non-linearity features in the wave formulas and the scattering process. E.D.R.Shearman^6^ proposed that sea waves of length not only Bragg wave, but also of *L* = *λ*, 3*λ*/2, 2*λ*, 5*λ*/2, 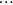, *etc*, which contains harmonics of Bragg wave, may take place Bragg resonant backscatter, and these harmonics correspond Doppler-shifted contributions should appear in the echo spectrum at 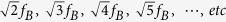. All of the above and other models^7^,^8^,^9^,^10^,^11^,^12^,^13^,^14^,^15^, were deduced from the scattering measurement data collected from actual sea surface based on Doppler effect, and has not been verified under man-made conditions.

It would be very difficult to collect the water wave scatter with a HF radar under man-made conditions due to the tens meters wave length of radio wave. Subscale experiment is helpful to understand the mechanism of the frequency shifts of radio wave backscatter from the Bragg and Non-Bragg water waves. A coherent UHF radar at 340 *MHz* is designed for the subscale experiment. Backscatter data from monochromatic Bragg water waves and non-Bragg water waves precisely generated by a man-made wave-tank was collected with the UHF radar. The spectrum of the radar data is analyzed, and frequency shift can’t be interpreted with the Doppler effect mentioned above. A new mechanism is thus proposed for the frequency shift of backscatter from Bragg and non-Bragg periodic gravity water waves.

## Wave tank experiment

**Figure 1:**
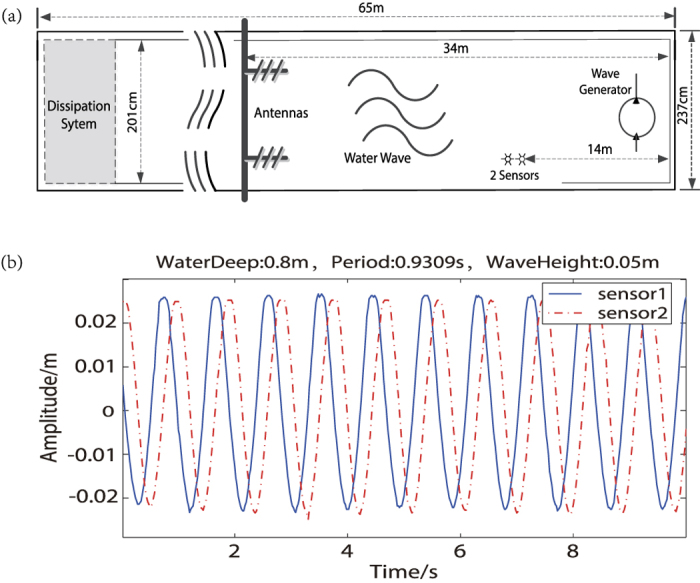
The experiment environment. (**a**)The wave tank and radar deployment. (**b**) The time domain waveforms of the water wave recorded with sensors.

The wave tank and UHF coherent radar position are shown in Fig. 1(a).

The coherent UHF radar was a full digital chirp radar^16^, operating at 340 *MHz* with bandwidth of 15 *MHz*. The sweep period was 0.043 *s*. Spectrum analyzer collected data of 256 sweep periods for coherent processing. It’s easy to see that the radar wave length *λ* is 0.88 *m* and the frequency resolution of the spectrum analyzer is 0.091 *Hz*. The Bragg wave length *λ*_*B*_ = *λ*/2 = 0.44 *m*. The transmitting and receiving antennas were V-V polarized, which were set on the left side of the tank. Radio wave beam was pointing to the water wave maker and the incidence angle to the water surface was about 3°.

The length, width, and the depth of the wave tank are 65 *m*, 201 *cm*, *and* 180 *cm* respectively. The wave-maker is on the right side, and the water wave propagates from right to the left as shown in Fig. 1(a). An energy dissipation system is on the left side, to prevent splash and water wave reflections. The wave tank generates periodic gravity sinusoidal waves, and the wave’s period ranges between 0.38 *s*~1.9 *s*. In order to keep the water wave shape and smooth, the amplitudes of the water wave changed with the wave length. Two pressure sensors, 0.4 *m* apart, with sample rate 50 *Hz*, were put in the water to monitor the water wave shape and parameters. Figure 1(b) demonstrates the time domain waveform recorded by the sensors, given the water wave length 3*λ*_*B*_, amplitude 5 *cm*, and the water deep 0.8 *m*. In term of Fig. 1(b), the wave length is 1.316 *m*, and the phase velocity is 1.43 *m*/*s*, which is almost exactly the same as the theoretical value 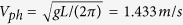.

## Experiment data and analyzing

Wave tank experiments were carried out to study the mechanism of Bragg and non-Bragg backscatter, i.e., the relationship between the frequency shift of the radar echo and the phase velocity of the water wave.

The wave length, frequency, amplitude of the periodic water wave generated by wave tank is shown in Table 1, and so are the phase velocities of the water waves, and the calculated Doppler shift according the phase velocities. *f*_*B*_ is the Bragg frequency, according to the radar wave length. which is 1.883 *Hz*.

**Figure 2:**
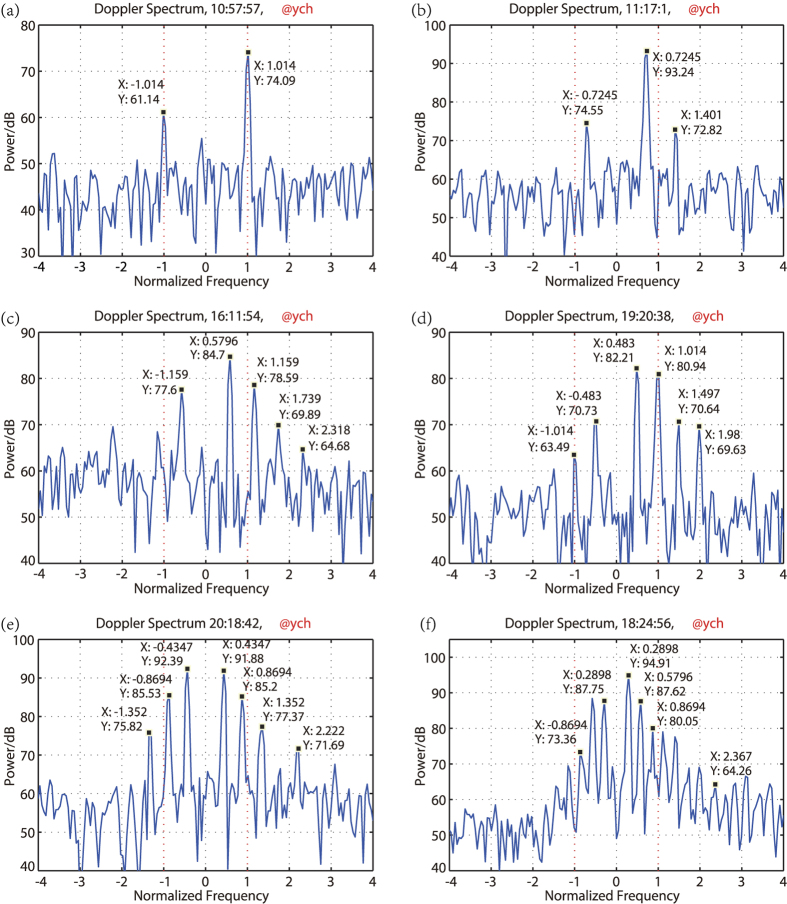
Power spectra of the backscatter from different wavelengths (**a**) *λ*/2, (**b**) 2*λ*/2, (**c**) 3*λ*/2, (**d**) 4*λ*/2, (**e**) 5*λ*/2, (**f**) 10*λ*/2.

The power density spectra of the backscatter from the wave tank collected by the UHF radar were shown in Fig. 2. These plots represent the received signal power against normalized frequency shift from the carrier (the carrier being located at zero, and the Bragg frequency at positions +1). As mentioned above, the frequency resolution of the spectra analyzer is 0.091 *Hz*, and the Bragg frequency is 1.883 *Hz*, so the normalized frequency resolution is 0.048.

Figure 2(a) is the power spectrum according to Bragg wave. Corresponding to the Table 1, the predicted Doppler shift *f*_*dpl*_ of the backscatter is the Bragg frequency *f*_*B*_, thus a strong peak should be at position +1 in the power spectrum Figure. In Fig. 2(a), the dominant peak is at the position 1.014. The discrepancy between the measured value and predicted value is less than the frequency resolution of the spectra analyzer. The result seems to confirm the Doppler effect in that the predicted Doppler shift is exactly the same as the measured shift. We should note, however, there is another peak at position −1.014, which can’t be interpreted by Doppler effect because there is no water wave with the wave length of *λ*_*B*_ propagating away from the radar.

For the water wavelength of 2*λ*_*B*_, the predicted Doppler shift of the backscatter, *f*_*dpl*_, should be 1.41 *f*_*B*_. The spectrum measured with the UHF radar is shown in Fig. 2(b). There is a peak at the position 1.401 indeed, but very weak. The dominant peak, in particular, is at the position 0.7245, about half the predicted Doppler shift *f*_*dpl*_. Opposite to the position, at position −0.7245, a secondary peak exists.

For the water wave length of 3*λ*_*B*_, the predicted Doppler shift *f*_*dpl*_ is 1.73 *f*_*B*_. The measured spectrum was shown in Fig. 2(c). The dominant peak is at the position 0.5796, about *f*_*dpl*_/3. Other peaks appear at positions about −*f*_*dpl*_/3, 2*f*_*dpl*_/3, *f*_*dpl*_, 4*f*_*dpl*_/3, *etc*.

Figure 2(d) is the measured spectrum of water wavelength 4*λ*_*B*_. The predicted Doppler shift *f*_*dpl*_ is 1.99 *f*_*B*_. The dominant peak is at the position about *f*_*dpl*_/4. Other peaks appear at positions about −*f*_*dpl*_/4, ±2*f*_*dpl*_/4, ±3*f*_*dpl*_/4, *and f*_*dpl*_ *etc*.

Figure 2(e) is the measured spectrum of water wavelength 5*λ*_*B*_. The predicted Doppler shift *f*_*dpl*_ is 2.21 *f*_*B*_. There are distinct peaks appear at positions about ±*f*_*dpl*_/5, ±2*f*_*dpl*_/5, ±3*f*_*dpl*_/5, *and f*_*dpl*_ *etc*. In contrast to other spectra mentioned, the strongest peak appears at minus frequency axis of position −*f*_*dpl*_/5.

Many other backscatter data of different water wavelengths were collected by coherent UHF radar. Figure 2(f) is the measured spectrum of water wavelength 10*λ*_*B*_. The predicted Doppler shift *f*_*dpl*_ of the backscatter should be 2.86 *f*_*B*_. There are distinct peaks appear at positions about ±*f*_*dpl*_/10, ±2*f*_*dpl*_/10, ±3*f*_*dpl*_/10, 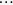, *etc*.

The following commonness can be reached by viewed the above experiment data: 1). Each of the spectra of the backscatter contains two or more peaks which are equally spaced; 2). The dominant peak is not the predicted Doppler effect peak corresponding to the waves phase velocity except when the wave is Bragg wave; 3). The water wave propagating to the radar results in not only positive frequency shift, but also the minus frequency shift, and the intensity of which may be dominant once in a while (e.g. Fig. 2(e)). The Doppler effect according to the water wave phase velocity can’t interpret the above phenomena of the frequency shift, and what is the mechanism? The radar’s range resolution of this experiment had been finite, and what characters would be present when the radar’s range resolution becomes infinite?

## Theoretical modeling

In order to reveal the mechanism of frequency shift of the backscatter, we study the backscatter of the sinusoidal water wave in time domain with finite radar range resolution.

Consider a parallel sinusoidal water wave propagating toward to the radar. Defining *xyz* rectangular coordinate system, the water surface is expressed as *ξ* = *ξ*(*x*, *t*), where *x* = (*x*, *y*) is horizontal coordinates, and *t* is the time. The radar is at position *x* = 0. The vector of radio wave 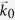 is of *x* axis positive direction.

Define the water depth *d*, the parameters of water wave are defined as vector 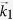, angle frequency *ω*_1_, amplitude *a*_1_. If 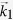 is of *x* axis positive direction, *k*_1_ > 0, it means the water wave propagating along the *x* axis positive direction. The range resolution of the radar is *D*. The water surface can be expressed as


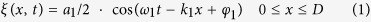


Ignoring the boundary effect, the backscattering coefficient *σ* can be expressed as *σ* = *σ*(*x*, *t*), and *σ*(*x*, *t*) is periodic both in space domain and time domain^17^. So, *σ*(*x*, *t*) can be written as *σ*(*x*, *t*) = *σ*(*x* + *mL*_1_, *t* + *mT*_1_), where *L*_1_ = 2*π*/*k*_1_, *T*_1_ = 2*π*/*ω*_1_, *m* is an integer.

Expansion of *σ*(*x*, *t*) in Fourier series,


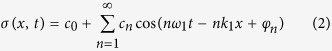


where *c*_*n*_ and *ϕ*_*n*_ are the amplitude and phase coefficient of the Fourier series. *c*_0_ can be neglected on account of it has no influence to the frequency shift.

Assume the incident electric field is


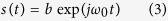


The electric field at the receiver scattering from position *x* is,


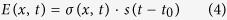


*t*_0_ = 2*x*/*c*, is the time delay of the radio wave, *c* is the velocity of light.

The average total electric field of the backscatter from radar range resolution 0~*D* is


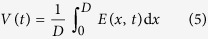


Substitute Eqs 2,3 and 4 into Eq. 5:


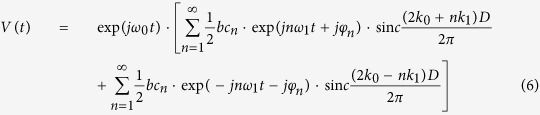


Or write Eq. 6 as:


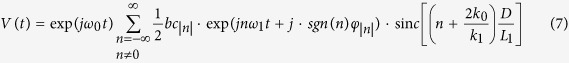


Define the angle frequency shift Δ*ω* = *ω* − *ω*_0_. In term of Eq. 7, the power spectrum of the backscatter during the coherent integrate time T can be written as:


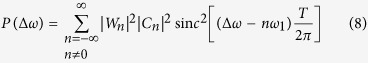



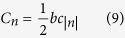



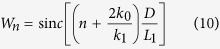


*n* is an integer, and 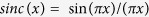.

When *T* → ∞,


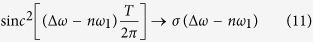


The parameter *T* is generally set to enough large in order to get high frequency resolution for the real ocean radar systems. So, Eq. 8 can be approximately rewritten as:


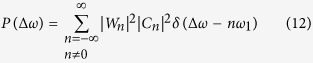


## Model Discussion

Equation 12 shows, that the power spectrum of the backscatter from periodic water wave is consisted of a series of discrete peaks, which are equally spaced in frequency axis by the fundamental frequency *ω*_1_, and the amplitude is decided by factor *C*_*n*_ and *W*_*n*_.

Consider a periodic sinusoidal water wave with wavelength of *L*_1_ = *N* · *λ*/2, propagating towards the radar. So, *k*_1_ = −2*k*_0_/*N*, and 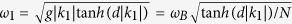. If the wave height *a*_1_ is very small comparing to the wave length *L*_1_, the backscatter model of the surface is close to a sinusoidal diffraction grating^5^,^12^,^17^,^18^, that means, *c*_1_ ≫ *c*_2_, *c*_3_, 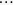 in Eq. 2. So, *C*_*n*_ is symmetric about *n* = 0, and with maximum value at *n* = ±1. *W*_*n*_ is a sinc function with symmetric center and maximum value at *n* = *N*.

**Figure 3:**
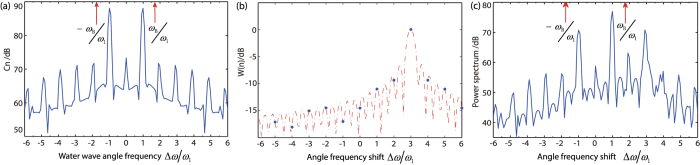
The relationship of *C*_*n*_, *W*_*n*_ and *P*(Δ*ω*). The amplitude of (**a**) *C*_*n*_, (**b**) *W*_*n*_, (**c**) *P*(Δ*ω*).

The relationship of *C*_*n*_, *W*_*n*_ and *P*(Δ*ω*) is illustrated in Fig. 3. Figure 3(a,b) are the simulated data *C*_*n*_ and *W*_*n*_ respectively with *k*_1_ = −2*k*_0_/3. Figure 3(c) is the power spectrum *P*(Δ*ω*). The relationship expressed in Eq. 12 can be seen as a filter with the input signal spectrum *C*_*n*_, transfer function *W*_*n*_, and output spectrum *P*(Δ*ω*). When the radar range *D* is a finite value, *W*_*n*_ acts as a band-pass filter, and the passband is highly influenced by the radar range resolution *D*. The smaller the *D*, the wider the passband. In Fig. 3(b), the maximum value of *W*_*n*_ occurs at the position 3*ω*_1_. After the *C*_*n*_ pass through *W*_*n*_, the value of *C*_*n*_ at position 3*ω*_1_ keeps undamped, and which at other frequency positions are depressed corresponding to the shape of |*W*_*n*_|.

For the experiment data, when *L*_1_ = *λ*/2, *k*_1_ = −2*k*_0_, we have *N* = 1 and *ω*_1_ = *ω*_*B*_. At position Δ*ω* = ±*ω*_1_, |*C*_*n*_| has the maximum value, and the symmetric center and maximum value of |*W*_*n*_| is at *Nω*_1_ = *ω*_1_, So the *P*(Δ*ω*) has a dominant peak at position *ω*_1_, and get a secondary peak at position Δ*ω* = −*ω*_1_ due to the lower sidelobe level of |*W*_*n*_| at this position. No obvious peaks at other positions can be seen because of 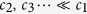, and 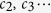 are depressed further by |*W*_*n*_|. The characters addressed above are clearly shown in Fig. 2(a). The two peaks are at ±1.014*ω*_*B*_, and there is a deviation of 0.014*ω*_*B*_ which is less than the frequency resolution 0.048*ω*_*B*_ of the radar system.

For the water wavelength of *L*_1_ = 2 · *λ*/2, *k*_1_ = −2*k*_0_/2, 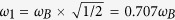. The maximum value of |*W*_*n*_| is at Δ*ω*_1_ = 2*ω*_1_. After the *C*_*n*_ pass through *W*_*n*_, component with frequency 2*ω*_1_ get maximum gain, and other components are depressed with different values. The experiment result is shown in Fig. 2(b). The actual frequency position is at ±0.7245*ω*_*B*_, 1.401*ω*_*B*_, and the frequency deviation is also less than the frequency resolution 0.048*ω*_*B*_ of the radar system.

Other instances include water wavelengths of *L*_1_ = 3 · *λ*/2 with *ω*_1_ = 0.576*ω*_*B*_, *L*_1_ = 4 · *λ*/2 with *ω*_1_ = 0.498*ω*_*B*_, *L*_1_ = 5 · *λ*/2 with *ω*_1_ = 0.442*ω*_*B*_, *L*_1_ = 10 · *λ*/2 with *ω*_1_ = 0.287*ω*_*B*_. The experiment data are in good accordance with the Eq.12.

From the experiment data, the main energy of the *W*_*n*_ is concentrated at the positive frequency axis due to the water wave propagating to the radar system. So for most of the power spectrum, *P*(*ω*_1_) is obviously great than *P*(−*ω*_1_) except Fig. 2(e). This is because that *W*_*n*_ is not a monotonic function.

When *D* → ∞, Eq. 10 changes to *W*_*n*_ → *σ*(*n* + 2*k*_0_/*k*_1_), then, the Eq. 12 can be written as:


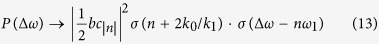


*W*_*n*_ is of non-zero value only when the 2*k*_0_/*k*_1_ is an integer. If the water wave propagating forward to the radar, *k*_1_ = −2*k*_0_/*N*,

The backscatter power spectrum of the water wave is


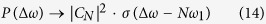


For the gravity water wave with wave number *k*_1_, 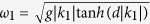, its phase velocity is 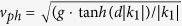, the Doppler shift according to the phase velocity is,


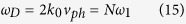


So,


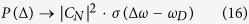


The Eq. 16 means, when the radar range trends to infinite, only those periodic water wave with the wave length integer multiple of the Bragg wave, can generate coherent backscatter, and the power spectrum contains only a single peak with a frequency shift Δ*ω* = *Nω*_1_, which just equals the Doppler shift corresponding to its phase velocity mathematically.

## Conclusion

Backscatter data from periodic sinusoidal water wave, both Bragg water wave and non-Bragg water waves precisely generated by a man-made wave tank, was collected with a coherent UHF radar. A few points can be observed from the spectrum of the backscatter data: 1) A single train gravity water wave propagating to the radar system can result in both positive frequency shifts and negative frequency shifts simultaneously; 2) The power spectrum of the backscatter from a gravity water wave with a fixed phase velocity contains a serial frequency shift components.

The above phenomena were obviously in conflict with the interpretation about the Doppler effect to the phase velocity of the gravity water wave.

A new mechanism was proposed to interpret the frequency shift of the backscatter from gravity water wave: The frequency shift is not the Doppler shift corresponding to the wave’s phase velocity, but the water wave’s frequency and its integral multiple with different weighting factor. The weighting factor is related with water wave length, radar wave length and radar range resolution.

Specifically, in the proposed model, when the radar range resolution trends to infinite, the frequency shift of backscatter from a periodic water wave just equals the Doppler shift corresponding to its phase velocity mathematically, but with different mechanism.

## Additional Information

**How to cite this article**: Wen, B. and Li, K. Frequency shift of the Bragg and Non-Bragg backscattering from periodic water wave. *Sci. Rep.* **6**, 31588; doi: 10.1038/srep31588 (2016).

## Figures and Tables

**Table 1:** Parameters of the water wave and calculated Doppler shift (*λ*_*B*_ = 0.44 *m*, *f*_*B*_ = 1.883).

Wave length *L*_1_	*λ*_*B*_	2*λ*_*B*_	3*λ*_*B*_	4*λ*_*B*_	5*λ*_*B*_	10*λ*_*B*_
Wave frequency *f*_*w*_ (*Hz*)	1.88	1.33	1.08	0.94	0.83	0.53
Wave amplitude *h* (*cm*)	1.0	4.0	5.0	10.0	15.0	20.0
Wave phase velocity *V*_*ph*_ (*m*/*s*)	0.83	1.17	1.43	1.65	1.83	2.37
Calculated Doppler shift *f*_*D*_	1.00 *f*_*B*_	1.41 *f*_*B*_	1.73 *f*_*B*_	1.99 *f*_*B*_	2.21 *f*_*B*_	2.87 *f*_*B*_
